# Observations on a Wound of the Right Eye-Brow, Followed by Loss of Sight in That Eye, and Amblyopy of the Left Eye

**Published:** 1817-04

**Authors:** M. Lamercier

**Affiliations:** Mayenne.


*For the London Medical and Physical Journal.*

### *Observations on a Wound of the right Eye-brow, followed by Loss of Sight in that Eye, and Amblyopy of the left Eye;* by M. Lamercier, M.D. of Mayenne.

JOHN DUVAL, domestic, æt. 32, of a sanguine temperament, tall, stout, and healthy, having gone to market with an ox, on the 16th Feb. 1816, holding him, he stooped down, and the ox suddenly throwing up his head, one of his horns struck Duval under the right eye-brow, and carried away with it three-fourths of the eye-brow, leaving the corona naked, and occasioning instantly almost an absolute loss of sight. On washing the wound, it was found that neither the ball of the eye nor the upper eye-lid were at all injured. Salt and water, and a compress of linen, were applied to stop the bleeding; and he was put to bed, where he rested three or four hours. On getting up, removing the bandage a little from off the left eye, he found that he could scarcely distinguish even with it the chairs and tables in the room; but, as he was in no pain, and even had an appetite, he took a bason of soup, and drank two glasses of cyder. Being unable to see his way home on foot, he procured a horse. In the evening he ate his supper as usual, went to bed early, and slept pretty well. The next morning, a woman of the village dressed his eye with a balsam, and, in three weeks, she perfectly healed the wound. He was then brought to consult me on the state of his sight.

I found an irregular cicatrix or scar protruding a little on the part next the eye; the upper eye-lid unhurt, and capable of motion, like the other; the globe of the eye without any sensible sensible alteration, simply black at bottom; the iris and the pupil motionless, and insensible to the flame of a candle. In broad day, the patient could not discover light from darkness. The left eye had no appearance of injury; the pupil only seemed more dilated than usual, but less so than that of the right eye; the iris sensible, and contracting visibly on raising the eye-lid, or on passing from a dark place into a strong light. The patient could only distinguish even large objects when near to him. In the day-time he would make a shift to go about alone, but, at night, only to places he was well acquainted with. He suffered no other inconvenience than the loss of sight; he ate, drank, and slept, as usual. After communicating my fears as to the total loss of sight in the right eye, and the hope of recovering that of the other, I ordered him, first, to apply four leeches round each eye; secondly, to take every three days during three weeks, two grains of antimonial tartrite of potass in two glasses of water, a table-spoonful every half hour; thirdly, the days that he did not take the emetic, to take an ounce of acidulated tartrite of potass in a little broth; fourthly, to wear, day and night, on the eyes dossils, (*sachets,*) moistened with a mixture of lime and muriat of ammonia, to be renewed every ten days at least; fifthly, to rub several times a day the circumference of the orbits with the balsam of Fioraventi; sixthly, to have a seton applied on the nape of the neck, and to keep it five or six weeks. Duval followed these directions, and, in six weeks, the sight of the left eye was completely restored; the right eye is entirely lost, open, but without deformity.

Even so early as the time of Hippocrates, it was known that wounds of the eye-brow sometimes produced loss of sight. Of the others who have written upon it, some attribute it to the disorganization or affection of the brain or its membranes; others, to extravasated blood in the cranium, or purulent deposits on the meninges, in the brain or on the beds of the optic nerves. Several assert, that it is owing to the lesion or irritation of the frontal or nasal branches. It is probable, that all of these may produce the effect; but, in the present case, the loss of sight proceeded only from the lesion of the frontal nerve. In fact, the patient felt no shock, he did not become insensible, nor even giddy, nor felt any pain except that arising from the wound. The loss of sight instantly followed the wound; from which it is evident, that it is the lesion of the frontal branch to which we must attribute the loss of sight; but, in what manner was this produced?—Was it by the paralysis of the muscles of the eye, or by that of the iris or the retina? M. Ribes has considered the question under these three points of view. He has demonstrated, in a luminous manner, that the paralysis of the iris, and of the muscles of the eye, do not prevent the retina from preserving the faculty of perceiving objects. It is, therefore, on this latter membrane that the lesion of the frontal nerve exerts its effect without denying the mediate influence, that the *trisplanchnique* (as M. Ribes thinks), may have in the paralysis of the retina, in consequence of the affection of the nerves of the eye-brow. It seems to me that one may conceive it, as Sabatier observes, by the medium of the nasal branch, which communicates with the lenticular ganglion, the latter furnishing the greater part of the ciliary nerves which have a direct and intimate relation with the medullary part of the retina, which unites itself with the ciliary body, and thereby receives the impression on the frontal branches. But how has the left eye participated the affection of the retina of the right eye in the present case? Is the retina isolated from the optic nerve, and different from it, as Winslaw, Bichat, and Ribes, seem to think? Or, are we to conclude, with Portal, Boyer, and Richerand, &c. with whose opinion I coincide, that it is only an expansion of the optic nerve? Following this latter opinion, may we conclude that the left eye becomes injured by the communication of the two optic nerves on the upper face of the sphenoid, or by the medium of the brain? and to assume, that, on account of the distance from the seat of the disorder, the effect is sensibly less, and, consequently, vision in the left eye was not destroyed, but only injured. Strictly speaking, we might thus explain the relation of the affection between the two optic nerves. But, when we reflect that the anatomical disposition is always the same, and that, in many cases, wounds of the eye-brow produce loss of sight in that eye without affecting the other, we are rather inclined to attribute the disorder of the other eye to the harmonic connexion between two organs, which have a parity of structure and similitude of functions, which are symmetrically situated in the same lateral division of the body, which make part of the same system, and have a reciprocal tendency to mutual affections, and to form, what in medicine we call, sympathy. In fact, experience has taught us, that, when one of the eyes is troubled in its functions, even without any apparent physical injury, the other soon becomes so. It is thus in neurology, when a nerve on one side suffers, it often happens that the corresponding nerve on the other side becomes painful. In pains in the two eyes, we may cure cure them by acting only on one. Several authors have differently explained this union and sympathy between corresponding organs, but hitherto nothing certain has been advanced upon the point, notwithstanding the positive manner of some physiologists.—Time and further experience may, perhaps, supply the desideratum.

